# Salivary flow rate in children and teenagers with Down syndrome: Systematic review and meta-analysis

**DOI:** 10.1590/1807-3107bor-2025.vol39.071

**Published:** 2025-09-08

**Authors:** Wilmer RAMÍREZ-CARMONA, Beatriz DÍAZ-FABREGAT, Antonio Hernandes CHAVES-NETO, Douglas Roberto MONTEIRO, Juliano Pelim PESSAN, Ana Cláudia de Melo Stevanato NAKAMUNE

**Affiliations:** (a) Universidade Estadual Paulista – Unesp, Araçatuba School of Dentistry, Department of Preventive and Restorative Dentistry, Araçatuba, SP, Brazil.; (b) Universidade Estadual Paulista – Unesp, Araçatuba School of Dentistry, Department of Basic Science, Araçatuba, SP, Brazil.; (c) Universidade do Oeste Paulista – Unoeste, Postgraduate Program in Health Sciences, Presidente Prudente, SP, Brazil.

**Keywords:** Adolescent, Chromosome Disorders, Disabled Children, Saliva, Salivary Glands, Systematic Review

## Abstract

The purpose of our review was to group the evidence and attempt to provide a consensus on the behavior of salivary flow rate in patients with Down syndrome. Observational studies evaluating salivary flow rate in children and teenagers with Down syndrome compared with non-syndrome individuals were selected. Ten sources of information were researched. The risk of bias was assessed by using the Newcastle Ottawa Scale tool . Inverse Variance was ty the SMD (95% Confidence Interval). The certainty of the evidence was determined according to the GRADE approach. Fourteen studies were evaluated. The results showed, with a very low certainty of evidence, that children and teenagers with Down syndrome present a lower salivary flow rate compared with non-syndrome controls (SMD: -1.71, 95%IC: -2.81; -0.60, p < 0.05), with significant differences in the saliva collection methods (p < 0.05) (Unstimulated saliva, SMD -5.07, 95%CI: -7.96; -2.18, p < 0.01; Stimulated saliva, SMD -0.80, 95%IC: -1.78; 0.17, p = 0.11). The behavior of the salivary flow rate is not significantly different between the age groups (p = 0.60) (up to 5 years old, SMD -1.85, 95%CI: -2.90; -0.81, p < 0.01; 2 to 18 years old, SMD -1.51, 95%CI: -2.24; -0.78, p < 0.01), and the sex (p = 0.70) (Male, SMD -1.77, 95%CI: -2.39; -1.16, p < 0.01; Female, SMD -1.53, 95%CI: -2.58; -0.48, p < 0.01). Children and teenagers with Down syndrome present a lower salivary flow rate with an unstimulated saliva collection method compared to non-syndrome.

## Introduction

Down syndrome is a chromosomal disorder derived from an effective trisomy of chromosome 21 meaning, so that instead of having 46 chromosomes, the person has 47. This condition is associated with intellectual disabilities and a wide variety of additional factors.^1^ Hypotonia, intellectual and learning disability, cervical instability, autism spectrum disorder, epilepsy, cerebrovascular disease, Alzheimer’s disease, and neuropsychiatric disease are the main clinical conditions of the neurological complications reported in Down syndrome patients.^2^

Evidence from studies has shown differences in parameters related to saliva and oral health in this population. For example, although there is no high-level scientific evidence to support the hypothesis that people with Down syndrome have a lower experience of dental caries,^3,4^ the possible trend towards a decrease in the caries index; This , In turn, is accompanied by common gingival and periodontal diseases.^5^ Children with Down syndrome have lower indices of pH, α-amylase, and of buffer capacity in the saliva, compared with control non-syndrome children.^6,7^ Furthermore, high levels of oxidative stress biomarkers, such as superoxide dismutase activity and malondialdehyde,^8^ and higher values of cytokines, such as IL-1β and IL8 are also reported in this population.^5^ Salivary parameters, especially salivary flow rate, seem to be a comparative factor in analyses.^6-8^

Saliva is a fluid with a complex composition, which results from the mixture of secretions from major and minor salivary glands, crevicular gingival fluid, microorganisms, desquamated epithelial cells, and food remains.^9^ Age, sex, collection method, medication use, pre-existing medical conditions, or diseases can affect salivary composition and flow rate.^10-12^

There is, however, no consensus in the scientific community about the influence of Down’s syndrome on salivary flow rate.^6-8,13^ Some authors have reported a significantly lower salivary flow rate in this population,^7,8^ while another study has stated that there was no difference^13^, or even that there is a tendency to a higher salivary flow rate in these patients.^6^ The proposal of our review was to group the evidence on the subject, and try to provide a consensus on the salivary flow rate behavior in children and adolescents with Down syndrome. The results of this review could help us clarify the salivary flow behavior in this population, especially as a risk factor for oral diseases.^3-5^ Thus, our review question is as follows: Is the salivary flow rate altered in children and teenagers with Down syndrome compared with non-syndrome controls?

## Methods

### Registration and protocol

This systematic review was carried out in accordance with the Preferred Reporting Items for Systematic Review and Meta-Analyses (Prisma) guidelines updated in 2020,^14^ and was registered under the CRD42022344856 (Prospero, https://www.crd.york.ac.uk/prospero) in July 2022.

### Eligibility criteria

Observational studies (cohort, case-control, and cross-sectional studies) evaluating salivary flow rate in children and teenagers with Down syndrome compared with non-syndrome controls were selected for this systematic review. The eligible population was children and adolescents (up to 18 years old), without sex restrictions; previously diagnosed with Down syndrome were considered to be the Exposure Group, while the comparison group (control?) consisted of the systemically healthy population without Down syndrome. The salivary flow rate was considered as the main outcome. Studies that assessed/performed salivary flow rate, but did not report the results, or results reported with other dichotomizations were excluded. Clinical registry studies and studies that were not concluded were not considered for inclusion. Parotid saliva alone was an exclusion criterion for our review. Moreover, children and teenagers with altered salivary flow rate due to conditions (except Down Syndrome) or medications/treatments that may be associated with a decrease or an increase in salivary flow rate were excluded. There were no restrictions in terms of language, year of publication of the study, ethnicity, or country.

### Information source

The sources of information were based on main databases such as PubMed / Medline, Scopus, Web of Sciences, Embase, and the Cochrane Library. Regional databases such as Lilacs and BBO accessed from the Virtual Health Library were also included. Open Grey (https://onlinelibrary.london.ac.uk/resources/databases/opengrey), Google Scholar (first 100 articles), and the Catalog of T*heses and dissertations of the Coordenação de Aperfeiçoamento de Pessoal de Nível Superior* (Capes) were assessed.

### Search strategy

The search strategy was elaborated according to the Population AND Exposure AND Outcome structure, for which mesh terms and entry terms were used. The data referring to the strategy used are described in Table 1. The date of the last search was 03/01/2024. The search strategy was validated from studies obtained in an exploratory search of the topic. The sensitivity of the search to identify all studies considered clearly eligible was high. The search process was carried out in pairs (researchers ACN and BDF). Any disagreements were settled by consensus with the help of a third investigator (WRC).

**Table 1:** Search strategy.

Search strategy	
#1	#1 MeSH descriptor: [Child, Preschool]
	#2 MeSH descriptor: [Child]
	#3 MeSH descriptor: [Adolescent]
	#4 (Preschool Child OR Children, Preschool OR Preschool Children OR Children OR Adolescents OR Adolescence OR Teens OR Teen OR Teenagers OR Teenager OR Youth OR Youths OR Adolescents, Female OR Adolescent, Female OR Female Adolescent OR Female Adolescents OR Adolescents, Male OR Adolescent, Male OR Male Adolescent OR Male Adolescents):ti,ab,kw
	#1 OR #2 OR #3 OR#4
#2	#1 MeSH descriptor: [Down Syndrome]
	#2 (Down Syndrome OR Syndrome, Down OR Mongolism OR Trisomy G OR Downs Syndrome OR Trisomy 21 OR Trisomy 21, Mitotic Nondisjunction OR Down Syndrome, Partial Trisomy 21 OR Partial Trisomy 21 Down Syndrome OR Trisomy 21, Meiotic Nondisjunction):ti,ab,kw
	#1 OR #2
#3	#1 MeSH descriptor: [Saliva]
	#2 (Saliva OR Salivas OR Salivary flow):ti,ab,kw
	#1 OR #2
#1 AND #2	
#1 AND #2 AND #3	

* Contact by email with a faculty where the study was presented to obtain the full text, and it was not available in the online version to make available. Diagram available from: Page MJ, McKenzie JE, Bossuyt PM, Boutron I, Hoffmann TC, Mulrow CD, et al. The PRISMA 2020 statement: an updated guideline for reporting systematic reviews. BMJ 2021;372:n71. doi: 10.1136/bmj.n71

### Selection process

The selection process was carried out in pairs in all phases. In the first phase, the studies were selected by title and abstract, and in the second phase, the full text was read according to the eligibility criteria. Any disagreements were settled by consensus. Articles in languages other than Spanish, Portuguese, and English were translated using the Google translator tool.

### Data collection process and data items

Data were collected by two independent reviewers (BDF and WRC) and verified by a third investigator (AH) who resolved possible disagreements. Data were collected on the authors, year of publication, country, study design, total number of participants, age, sex, characteristics of the children, saliva collection methods, salivary flow rate, funding, limitations or bias reported, and conflict of interest.

### Study risk of bias assessment

The risk of bias was assessed by using the Newcastle Ottawa Scale tool (NOS) modified for cross-sectional studies.^15^ This tool assesses the selection, comparability, and outcome process according to bias for these types of studies. This process was carried out by two researchers in pairs and individually (BDF and WRC), and doubts or disagreements were resolved by consensus. Regarding the risk of bias, individual studies were assessed as low risk (≥ 7 stars) or high risk (< 7 stars).^16^

### Effect measures

The salivary flow rate (mean and standard deviation) was evaluated as a continuous variable, expressed in mL of saliva (stimulated or unstimulated) per minute (mL/min). The measure evaluated was the Standardized Mean Difference (SMD) between the exposed and control groups, due to the variability of saliva collection methods. The data provided in median and ranges were transformed into mean and standard deviation.^17^

### Synthesis methods

The Standardized Mean Difference was measured using Inverse Variance (IV) as the statistical method, with a 95% confidence interval (CI). The chi-square test and I^2^ statistic were used to assess the heterogeneity in the studies (p < 0.10). The overall effect was assessed using the Z statistic at a 5% significance level. The Tau^2^ was used to interpret the data variability of effect size, and the analysis model was carried out using the random effect model^18^. In cases of high heterogeneity in the results, this was explored with subgroup analysis considering saliva collection methods (stimulated or unstimulated), age of participants (years old), and sex (male or female). The analysis was adjusted by risk of bias (low risk of bias). Statistical analyses were performed in Review Manager version 5.4.

### Publication bias assessment

Small study effects and publication bias were assessed by Egger’s test (p < 0.10) for pooling up to 10 studies, and the funnel plot graph.^19^ The ‘trim and fill’ method was performed to identify and correct for funnel plot asymmetry arising from publication bias.^20,21^ Statistical analyses were carried out in R version 4.1.3.

### Certainty of the evidence assessment

The certainty of evidence was determined according to the Grading of Recommendations Assessment, Development, and Evaluation (GRADE) approach.^22^ Cross-sectional studies start with low evidence. Parameters such as the risk of bias, inconsistency, indirectness, imprecision, publication bias, effect size, absence of confounding factors, and dose-response effect were assessed for classification.^23^ The certainty of the evidence was classified as very low, low, moderate, or high. GRADEpro software (gradepro.org) was used to perform the analysis and to generate the results.

## Results

### Study selection

**Figure 1:**
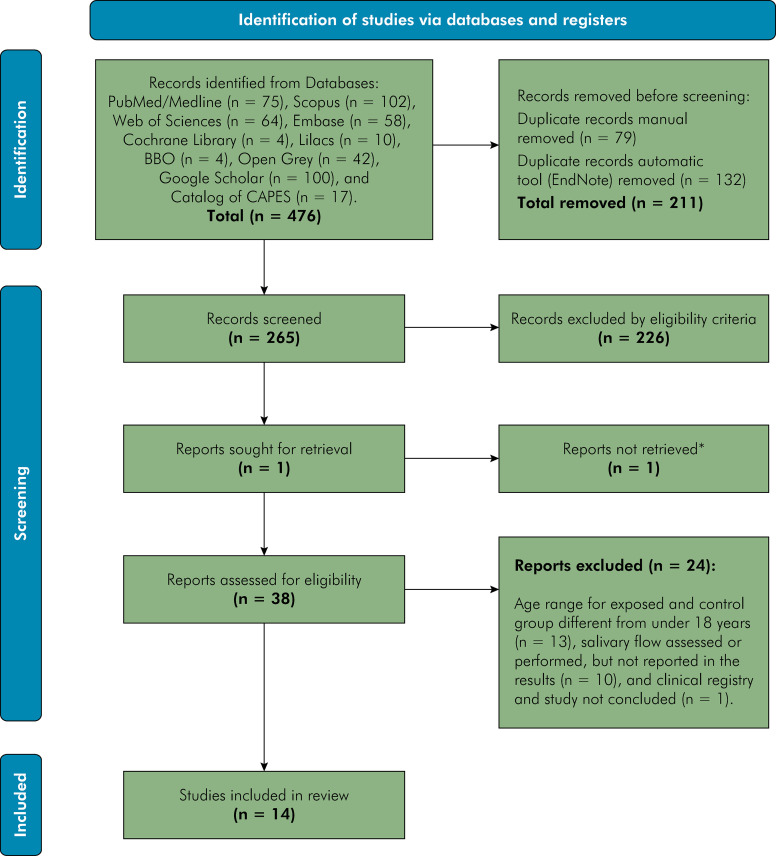
Flow-Diagram.

A total of 500 studies were identified from the databases, as follows: PubMed / Medline (n = 83), Scopus (n = 107), Web of Sciences (n = 73), Embase (n = 63), Cochrane Library (n = 8), Lilacs (n = 7), BBO (n = 1), Open Grey (n = 56), Google Scholar (n = 100), and CAPES Catalog (n = 2). Subsequently, 240 duplicate studies were removed and 220 other studies were excluded according to the eligibility criteria. Of the 39 eligible studies, 25 studies were excluded for the following reasons: age range for exposed and control groups different from under 18 years; salivary flow rate assessed or performed, but not reported in the results; results reported with other dichotomizations; clinical registry study, and study not concluded. Finally, 14 clinical studies were included in the current review (Figure 1).

### Study characteristics and individual results

This systematic review included 14 studies with a cross-sectional design. The studies were conducted in developed and underdeveloped countries on four continents, with seven in Brazil and one each in the Kingdom of Saudi Arabia, Portugal, Indonesia, New Zealand, Türkiye, Iraq, and India, respectively. The studies evaluated a total of approximately one thousand children and adolescents. Samples or records of participants with Down syndrome were obtained from centers and schools for special children, dental clinics, pediatric hospitals, the Association of Parents and Friends of Exceptional People, and national databases. Controls were siblings or healthy children and teenagers from preschools and elementary schools, dental clinics, and hospitals in the same city or region. A wide variety of saliva collection methods were used. The individual results are described (Table 2).

**Table 2:** Characteristics of studies included.

Study identification	Country	Study design	Participants	Characteristics of Children and adolescents	Saliva collection methods	Salivary flow rate (mL/min)	Funding	Limitations or bias reported	Conflict of interest
						Mean (standard deviation)			
Alzughaibi, Filimban, & Arafa, 2017^6^	Kindom of Saudi Arabia	Cross-sectional study	DS: 100 children and adolescents	DS: Special needs centers	Unstimulated saliva	0.80 (0.66)	Faculty of Dentistry in Umm Al-Qura University.	Does not justify the sample size. It does not describe in detail, how the evaluation process was performed.	None
				Areias et al., 201230	Time: 9 to 11 am				
				Bachtiar, Salmiah, & Luthfiani, 20187	Fasting: 2 h (drink and food)	Control: 0.64 (0.45)			
			Control: 103 children and adolescents	Control: Non-syndrome children and adolescents from elementary and kindergarten schools		p < 0.05			
			Age: 4 to 15 years old						
			Sex: Matched between groups						
Areias et al., 2012 ^30^	Portugal	Cross-sectional study	DS: 44 children and adolescents	Portuguese national database	Stimulated saliva	0.30 (0.24)	The Faculty of Dental Medicine of the University of Porto, Portugal.	Does not justify the sample size. No detailed description provided of how the evaluation process was performed	None
					Stimulus: Masticatory (paraffin)				
					Time: 8 am	Control: 0.47 (0.29)			
					Fasting: 2 h (eating, tooth brushing, or mouth washing)				
			Control: 44 children and adolescents	Control: The sibling		p < 0.05			
				DS: closest in age who was living in the same household was used as the matched control.					
			Age: 6 to 18 years old						
			Sex: Not reported						
Bachtiar, Salmiah, & Luthfiani, 2018^7^	Indonesia	Cross-sectional study	30 children and adolescents (17 males and 13 females)	Medan Helvetia and Medan Timur district	Unstimulated saliva	DS: 0.35 (0.13)	No reported	Does not justify the sample size. No detailed description provided of how the evaluation process was performed	None
	
					Time: 9 to 11 am				
						Control: 0.47 (0.17)			
					Fasting: Not reported				
			Control: 30 children and adolescents (17 males and 13 females)	Control: Non-syndrome children and adolescents. (Medan Helvetia and Medan Timur district)		p < 0.05			
			Age: 6 to 18 years old						
Balaji et al., 2016 ^13^	New Zealand	Cross-sectional study	DS: 19 children and adolescents	DS: Participants were contacted through the Pediatric Dentistry Clinic	Unstimulated saliva (Preweighed Salivette ^®^)	DS: 1.10 (0.50)	New Zealand Dental Association and Ministry of Health	Does not justify the sample size. Fewer participants in the experimental group than the minimum necessary. No description of the sampling strategy. It does not describe how the evaluation process was in detail.	None
					Time: Not reported				
					Fasting: Not reported	Control: 1.0 (0.30)			
			Control: 10 children and adolescents	Control: Sibling of closest age.		p > 0.05			
			Age: 2 to 12 years old						
			Sex: Matched between groups						
Cogulu et al., 2006 ^31^	Türkiye	Cross-sectional study	DS: 73 children and adolescents (38 males and 35 females)	DS: Department of Pediatrics	Stimulated saliva	DS:	None	Does not justify the sample size. No detailed description provided of how the evaluation process was performed	None
						1.05 (0.17)			
					Stimulus: Masticatory (paraffin)				
					Time: 9 to 12 am	Control: 1.04 (0.17)			
					Fasting: 2 h (eat or drink)				
			Control: 70 children and adolescents (33 males and 37 females)	Control: Non-syndrome children and adolescents.		p > 0.05			
				(State schools in Izmir)					
			Age: 7 to 12 years old						
Domingues et al., 2017 ^8^	Brazil	Cross-sectional study	DS: 18 children and adolescents (8 male and 10 female)	DS: APFE	Stimulated saliva	DS: 0.25 (0.10)	Brazilian Financing Agency FAPESP (São Paulo Research Foundation––grant 2013/18010-2) and the Association of Parents and Friends of People with Disabilities (APAE) in Araraquara.	Fewer participants in the experimental group than the minimum necessary. No detailed description provided of how the evaluation process was performed	None
					Stimulus: Masticatory (paraffin)				
					Time: 9 to 11 am	Control: 0.63 (0.23)			
					Fasting: 2 h (eat)				
			Control: 23 children and adolescents (9 males and 14 females)	Control: Non-syndrome children and adolescents (Pediatric Clinic)		p < 0.05			
			Age: 6 to 12 years old						
Habibe et al., 2020 ^5^	Brazil	Cross-sectional study	DS: 22 children and adolescents	DS: AFPE	Unstimulated saliva (Preweighed Salivette ^®^)	DS: 0.28 (0.23)	São Paulo Research Foundation (Fundação de Amparo à Pesquisa do Estado de São Paulo, FAPESP), National Council for Scientific and Technological Development (CNPq) and National Institute of Science and Technology in (CNPq/CAPES/FAPEMIG). Theranostics and Nanobiotechnology.	Does not justify the sample size. No detailed description provided of how the evaluation process was performed	None
								Convenience samples.	
					Time: Not reported				
						Control: 0.36 (0.14)			
					Fasting: 1 h (eating, drinking liquids or brushing their teeth)				
			Control: 22 children and adolescents	Control: Non-syndrome children and adolescents		p < 0.05			
			Age: 7 to 18 years old						
			Sex: Matched between groups						
Jafer, 2009 ^24^	Iraq	Cross-sectional study	DS: 50 children	DS	Stimulated saliva	DS: 0.47 (0.08)	None	No detailed description provided of how the evaluation process was performed	None
					Stimulus: Not reported*				*contact by email with a faculty where the study was presented to obtain the full text, and it was not available in the online version to make available.
						Control:			
					Time: Not reported*	0.92 (0.65)			
					Fasting: Not reported*				
						p < 0.05			
			Control: 50 children	Control: Non-syndrome children					
			Age: 7 to 10 years old						
			Sex: Not reported*						
Raurale et al., 2013 ^32^	India	Cross-sectional study	DS: 30 children and adolescents	DS: Hospital Ahmednagar	Unstimulated saliva	DS: 0.30 (0.03)	None	Does not justify the sample size. No detailed description provided of how the evaluation process was performed	None
					Time: 8 to 10 am	Control: 1.26 (0.05)			
					Fasting: Not reported				
			Control: 30 children and adolescents	Control: Non-syndrome children and adolescents		p > 0.05			
			Age: 8 to 14 years old						
			Sex: Not reported						
Schutz et al., 2013 ^25^	Brazil	Cross-sectional study	DS: 30 adolescents	DS: Down’s syndrome diagnosed. (City special schools)	Stimulated saliva	DS: 0.32 (0.25)	None	Does not justify the sample size. No description of the sampling strategy No detailed description provided of how the evaluation process was performed	None
					Stimulus: Masticatory (latex)				
	
					Fasting: 2 h (eat)				
			Control: 30 adolescents	Control: Non-syndrome adolescents		p < 0.05			
			Age: 10 to 15 years old						
			Sex: Matched between groups						
Siqueira & Nicolau, 2002 ^26^	Brazil	Cross-sectional study	DS: 17 children (8 males and 9 females)	DS: APFE	Stimulated saliva	DS: 0.38 (0.15)	Conselho Nacional de Desenvolvimento Científico e Tecnologico (CNPq).	Does not justify the sample size. No description given of the sampling strategy. No detailed description provided of how the evaluation process was performed	None
					Stimulus: Masticatory (paraffin)	Male: 0.41 (0.19)			
						Female: 0.36 (0.13)			
					Time: 9 to 11 am	Control: 0.88 (0.26)			
					Fasting: 2 h (eat)				
						Male: 0.93 (0.25)			
						Female: 0.82 (0.27)			
						p < 0.05			
			Control: 18 children (9 males and 9 females)	Control: Non-syndrome children					
			Age: 6 to 10 years old						
Siqueira et al., 2004 ^27^	Brazil	Cross-sectional study	DS: 22 children (12 males and 10 females)	DS: APFE	Stimulated saliva	DS: 0.37 (0.13)	Conselho Nacional de Desenvolvimento Científico e Tecnologico (CNPq).	Does not justify the sample size. No description of the sampling strategy. It does No detailed description provided of how the evaluation process was performed	None
					Stimulus: Masticatory (paraffin)				
						Male: 0.41 (0.17)			
						Female: 0.35 (0.19)			
					Time: 8 to 10 am				
						Control: 0.95 (0.21)			
					Fasting: Not reported				
						Male: 0.97 (0.36)			
						Female: 0.84 (0.25)			
			Control: 21 children	Control: Non-syndrome children		p < 0.05			
			(11 males and 10 females)						
			Age: 6 to 10 years old						
Siqueira et al., 2005 ^28^	Brazil	Cross-sectional study	DS: 25 children (11 males and 14 females)	DS: APFE	Unstimulated saliva	DS: 0.34 (0.14)	Not reported	Does not justify the sample size. No description of the sampling strategy. No detailed description provided of how the evaluation process was performed.	None
					Time: 9 to 10 am	Male: 0.37 (0.14)			
						Female: 0.32 (0.19)			
					Fasting: 2 h (eat)				
						Control: 0.56 (0.18)			
						Male: 0.63 (0.22)			
						Female: 0.47 (0.27)			
			Control: 21 children (10 males and 11 females)	Control: Non-syndrome children		p < 0.05			
			Age: 2 to 60 months						
Siqueira et al., 2007 ^29^	Brazil	Cross-sectional study	DS: 20 children	DS: APFE	Unstimulated saliva (using slight suction through a soft plastic catheter)	DS: 0.32 (0.10)	CAPES (Brazilian Ministry of Education)	Does not justify the sample size. No description of the sampling strategy. No detailed description provided of how the evaluation process was performed	None
					Time: 9 to 10 am	Control: 0.58 (0.11)			
					Fasting: 2 h (eat)				
						p < 0.05			
			Control: 18 children	Control: Non-syndrome children. (Dental Clinic)					
			Age: 12 to 60 months						
			Sex: Not reported						

DS: Down’s syndrome diagnosed; AFPE: Association of Parents and Friends of Exceptional.

### Risk of bias in studies

The studies were evaluated according to the risk of bias; 8 were classified as high risk of bias^5,13,24-29^, and 6 as low risk of bias^6-8,30-32^ (Table 3). The main biases detected in the selection of the participants were no justification of the sample size^5-7,13,25-29,30-32^; no description of the sampling strategy;^13,25-29^ and convenience samples^5^. Regarding the comparability between the groups, the majority of the studies were comparable in the characteristics between them, except two studies^8,13^ that had fewer participants in the experimental group than the minimum necessary. According to the outcome assessed, none of the studies described the evaluation process in detail, for example, if the person who collected the saliva was the same one who evaluated it and knew which group the participant was from, or if a code was created to carry out the blinding, etc.

**Table 3:** Risk of bias in individual studies.

Study identification	Selection	Comparability	Outcome	Total	Risk of bias
	(5 stars)	(2 stars)	(3 stars)	(10 stars)	
Alzughaibi, Filimban, & Arafa, 2017^6^	4:00 AM	2	1 ^b^	7	Low risk of bias
Areias et al., 2012 ^30^	4:00 AM	2	1 ^b^	7	Low risk of bias
Bachtiar, Salmiah, & Luthfiani, 2018^7^	4:00 AM	2	1 ^b^	7	Low risk of bias
Balaji et al., 2016 ^13^	3 ^a, c^	1 ^d^	1 ^b^	5	High risk of bias
Cogulu et al., 2006 ^31^	4:00 AM	2	1 ^b^	7	Low risk of bias
Domingues et al., 2017 ^8^	5	1 ^d^	1 ^b^	7	Low risk of bias
Habibe et al., 2020 ^5^	3 ^a, f^	2	1 ^b^	6	High risk of bias
Jafer, 2009 ^24^	3 ^g^	2	1 ^b^	6	High risk of bias
Raurale et al., 2013 ^32^	4:00 AM	2	1 ^b^	7	Low risk of bias
Schutz et al., 2013 ^25^	3 ^a, c^	2	1 ^b^	6	High risk of bias
Siqueira & Nicolau, 2002 ^26^	3 ^a, c^	2	1 ^b^	6	High risk of bias
Siqueira et al., 2004 ^27^	3 ^a, c^	2	1 ^b^	6	High risk of bias
Siqueira et al., 2005 ^28^	3 ^a, c^	2	1 ^b^	6	High risk of bias
Siqueira et al., 2007 ^29^	3 ^a, c^	2	1 ^b^	6	High risk of bias

The risk of bias was assessed by using they Newcastle Ottawa Scale (NOS) , modified version for cross-sectional studies. Regarding the risk of bias, individual studies were assessed as low risk (≥ 7 stars) or high risk (< 7 stars). a does not justify the sample size. b No detailed description provided about how the evaluation process was performed ,Flow- c No description of the sampling strategy. d Fewer participants in the experimental group than the minimum necessary. f Convenience samples. g Contact by email with a faculty where the study was presented to obtain the full text and no answers.

### Results of syntheses

**Figure 2:**
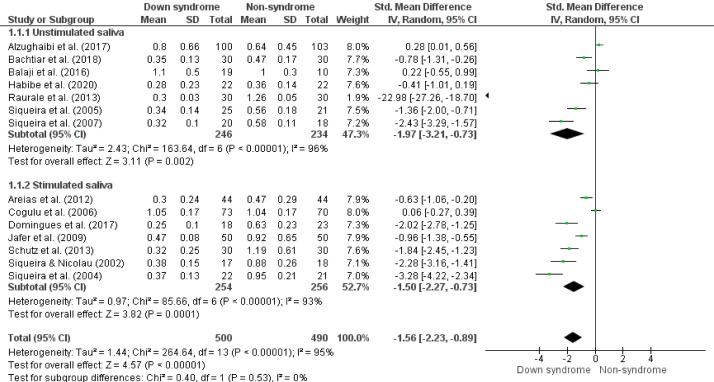
Forest plot comparing the difference in salivary flow rate (mL/min) between groups of children and adolescents with Down syndrome and non-syndrome by saliva collection method.

**Figure 3:**
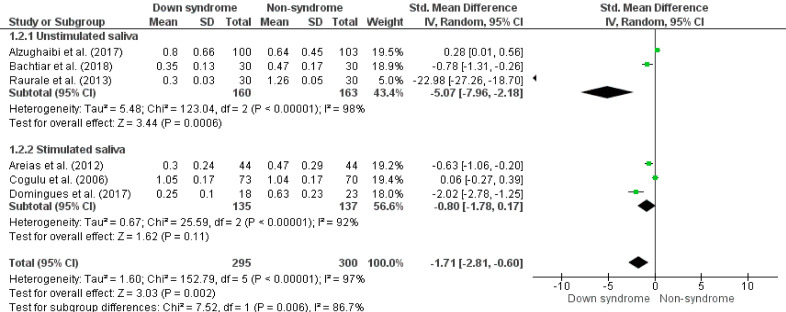
Forest plot comparing the difference in salivary flow rate (mL/min) between groups of children and adolescents with Down syndrome and non-syndrome by saliva collection method and adjusted by risk of bias.

**Figure 4:**
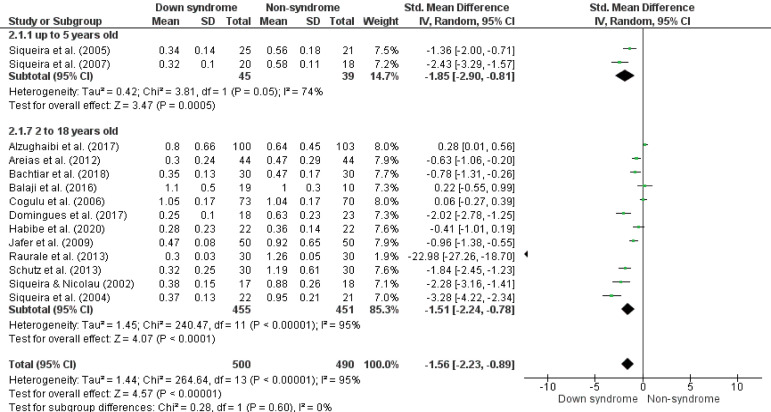
Forest plot comparing the difference in salivary flow rate (mL/min) between groups of children and adolescents with Down syndrome and non-syndrome by age.

**Figure 5:**
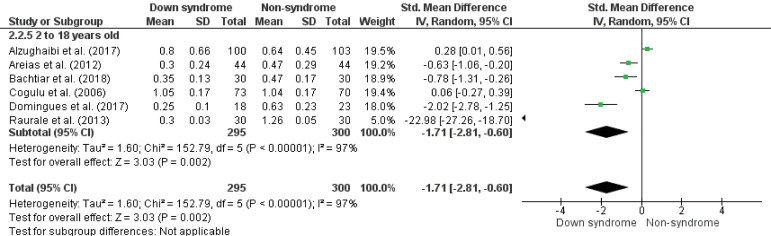
Forest plot comparing the difference in salivary flow rate (mL/min) between groups of children and adolescents with Down syndrome and non-syndrome by age and adjusted by risk of bias.

**Figure 6:**
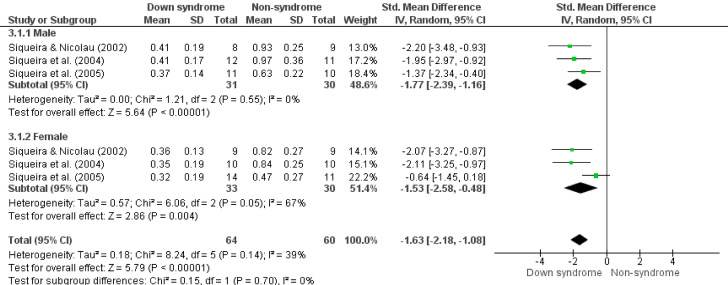
Forest plot comparing the difference in salivary flow rate (mL/min) between groups of children and adolescents with Down syndrome and non-syndrome by sex.

The meta-analysis showed that children and teenagers with Down syndrome have a lower salivary flow rate compared to non-syndrome (SMD -1.56, 95%CI -2.23; -0.89, p < 0.05, I^2^95%) (Figure 2). When adjusted by low risk of bias, the results are similar (SMD -1.71, 95%CI -2.81; -0.60, p < 0.05, I^2^ 97%), but with significant differences (p < 0.01) in behavior between the subgroups of the saliva collection methods (Unstimulated saliva, SMD -5.07, 95%CI -7.96; -2.18, p < 0.01, I^2^98%; Stimulated saliva, SMD -0.80, 95%CI -1.78; 0.17, p = 0.11, I^2^92%) (Figure 3). In the results by age groups, significant differences were not observed in the salivary flow rate behavior between the ages (p = 0.60) (up to 5 years old, SMD -1.85, 95%CI -2.90; -0.81, p < 0.01, I^2^74%; 2 to 18 years old, SMD -1.51, 95%CI -2.24; -0.78, p < 0.01, I^2^95%) (Figure 4). In the same way, similar results were observed when adjusting for the risk of bias (2 to 18 years old, SMD -1.71, 95%CI -2.87; -0.60, p < 0.01, I^2^97%) (Figure 5). In addition, the male and female sexes presented similar behavior in salivary flow rate between the subgroups (p = 0.70), being lower in the experimental group (SMD -1.63, 95%CI -2.18; -1.08, p < 0.05, I^2^39%) (Male, SMD -1.77, 95%CI -2.39; -1.16, p < 0.01, I^2^0%; Female, SMD -1.53, 95%CI -2.58; -0.48, p < 0.01, I^2^67%) (Figure 6).

### Reporting biases

The funnel plot graph showed visually detected asymmetry, and after performing the Egger’s test, publication bias was corroborated (p < 0.01) (Figure 7.1). The analysis was adjusted by the ‘trim and fill’ method to estimate missing studies due to publication bias (p = 0.74) (Figure 7.2).

**Figure 7:**
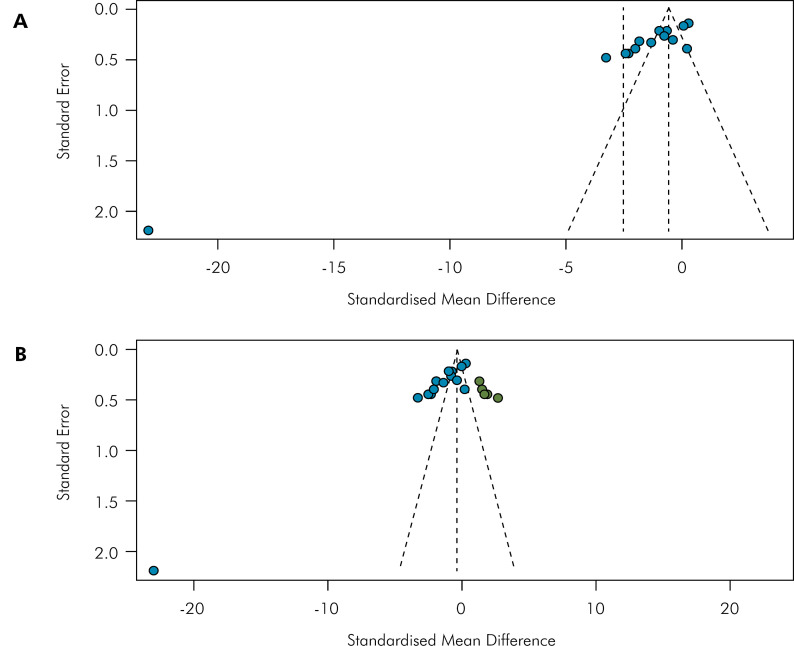
(A). Standard Error (*x* axis) and Standardized Mean Difference (*y* axis). Funnel plot shows an asymmetry detected. Egger test (t = -6.60, df = 12, p-value < 0.0001). (B). Standard Error (*x* axis) and Standardized Mean Difference (*y* axis). Funnel plot shows publication bias adjusted by the ‘trim and fill’ method. Egger test (t = -0.33, df = 18, p-value = 0.7430). Fill points estimate the number of missing studies.

### Certainty of evidence

Regarding the main outcome, the certainty of the evidence of the results was very low, due principally to the risk of bias (sample size), high heterogeneity, imprecision, and the design of the studies (cross-sectional). According to analysis by sex, the evidence was very low due to the low number the studies and participants and the high risk of bias (Table 4).

**Table 4:** The certainty of the evidence assessed by using the Grading of Recommendations Assessment, Development and Evaluation (GRADE) approach.

Outcome	Study design (n)	Risk of bias	Inconsistency	Indirectness	Imprecision	Other considerations	Certainty	Explanations
Salivary flow rate (See Figure 2 and 4)	Cross-sectional studies (n = 14)	very serious^1^	very serious^2^	not serious	serious^3^	Publication bias^4^. Dose-response gradient was not assessed. Confounding factors were evaluated^5^. Very large effect (SMD > 0.8 or SMD < -0.8).	⨁◯◯◯	^1^ More than half of the studies were high risk of bias (See Table 4).
							Very low	^2^ High heterogeneity due to the age ranges of participants verified by subgroup analysis (See Figure 4).
								^3^ Total number of people evaluated was 990, and Confidence Interval (CI) more than CI ± 0.5.
								^4^ Publication bias detected (See Figure 7).
								^5^ Subgroups analysis (saliva collected methods, age and risk of bias)
Salivary flow rate adjusted by low risk of bias (See Figure 3 and 5)	Cross-sectional studies (n = 6)	not serious	very serious^6^	not serious	serious^7^	Publication bias not applicable (n < 10). Dose-response gradient was not assessed. Confounding factors were evaluated^8^. Very large effect (SMD > 0.8 or SMD < -0.8).	⨁◯◯◯	^6^ High heterogeneity due to the age ranges of participants verified by subgroup analysis (See Figure 5).
							Very low	^7^ Total number of people evaluated was 595, but CI more than ± 0.5.
								^8^ Subgroups analysis (saliva collected methods)
Salivary flow rate by sex (See Figure 6)	Cross-sectional studies (n = 3)	very serious^9^	not serious^10^	not serious	very serious^11^	Publication bias not applicable (n < 10). Dose-response gradient was not assessed. Confounding factors were evaluated^12^. Very large effect (SMD > 0.8 or SMD < -0.8).	⨁◯◯◯	^9^ All studies were high risk of bias (See Table 4).
							Very low	^10^ Low heterogeneity
								^11^ Total number of people evaluated was 124. CI more than ± 0.5.
								^12^ Subgroups analysis (only by sex)

## Discussion

The results of this meta-analysis suggested that the salivary flow rate was lower in patients with Down syndrome, but that there were differences between collection methods. The quantity of non collected was lower in individuals with Down syndrome, while the stimulated saliva collected showed no differences.

Saliva is secreted by three pairs of major salivary glands, the parotid, submandibular, and sublingual, by a pair of tubarial glands located in the oropharynx, and by hundreds of minor salivary glands.^33^ Control of the secretions of these glands involves afferent pathways for conducting signals to the central nervous system, efferent pathways of the autonomic, sympathetic, and parasympathetic nervous systems, responsible for conducting signals to the glands. In the absence of stimuli and the presence of the individual’s awareness, salivary secretion is controlled by minor reflex activities of the central nervous system.^34^

The volume of saliva produced under these conditions as a function of time in minutes is called resting, basal, or unstimulated salivary flow.^35^ The submandibular, sublingual, parotid, and minor glands contribute to this flow. The salivary flow produced in response to the stimulus of chemo and/or mechanoreceptors is called stimulated. The responses of different types of glands to stimuli differ, with the parotid glands being more strongly stimulated by mastication, and the submandibular and sublingual glands being more responsive to gustatory stimuli. Whereas the smaller glands flow practically continuously , but are influenced by the circadian rhythm.^34^ Unstimulated salivary flow is more important for assessing glandular function, as changes in function do not always affect unstimulated flow.^36^

The literature has shown conflicting data regarding salivary flow rate in patients with Down syndrome, when compared with non-syndrome individuals. Part of the discrepancies result from the fact that some studies were conducted with unstimulated saliva and others with flow, mostly after mechanical stimulation. The unstimulated flow varies as a function of the health status and physiological conditions of individuals^35^. The submandibular and sublingual glands contribute to approximately 75% of this flow, while the parotid glands account for 15–20% and the minor glands for 5–8%. The reduction in unstimulated salivary flow seen in Down syndrome may result, at least in part, from the decreased reflex activity of the central nervous system^34^ or the difficulty of spitting in this population. The absence of a significant difference in the stimulated salivary flow rate of individuals with Down syndrome compared with non-syndrome individuals suggests that the lower mastication force, due to the reduction in electrical potential in muscles involved in this process,^37^ tongue hypotonia, and temporomandibular disorders^38^ common in the syndrome. These factors do not interfere with the secretory capacity of the parotid glands, which contribute to 50% of the mechanically stimulated salivary flow.^9,39,40^ In contrast,, it should be considered that this population, as well as the child population under 5 years of age, have difficulties with spitting, so methods such as light suction through a soft plastic catheter or other device would facilitate collection and are recommended methods.^29^

The electrolyte concentration gradient, maintained by the active transport of sodium and potassium, in combination with the polarized distribution of membrane transport proteins also influences the secretion of fluids by acinar cells of the salivary glands, and so does the modification of these glands by the duct cells.^41^ There is no evidence, however, that the reduction in salivary flow rate after mechanical stimulation in patients with Down syndrome results from the higher sodium concentration observed in these conditions.^26,27^

The time when the saliva samples were obtained may have interfered with the results, as salivary secretion is influenced by the circadian rhythm.^34^ To eliminate this possible bias, all studies included in the review reported sample collection in the morning. In addition, age can interfere with salivary gland secretion^39^ and people with Down syndrome may experience accelerated neurological aging^42^ caused by an increase in the oxidative state,^43^ so analyses were carried out to assess the impact of these biases on the results. From birth to adolescence, there are variations in the composition of saliva that reflect the development and maturation of the salivary glands^44,45^ and these factors can be associated with the different stages of dentition; i.e., deciduous, mixed, and permanent dentition.^46,47^ It is known that unstimulated salivary flow is higher at 30 and 42 months of age than at 18 months^48^ and decreases throughout life.^40,43,49^ With aging, there is a reduction in the glandular secretory epithelium, an increase in intraglandular lipid and connective tissue deposits, and a reduction in blood perfusion.^50^ Moreover, all of these changes compromise the salivary flow, whether stimulated or not. The studies included in this review collected saliva from a broad age groups, and it was not possible to perform subdivisions for data analysis with the lower range of ages. Despite this, we were able to verify that the salivary flow was lower in patients with Down syndrome, irrespective of age.

Sex is another factor that can interfere with salivary flow results. Lower unstimulated flow values have been described in females compared with males in different age groups.^46^ This difference is attributed to the smaller size of the salivary glands in women,^51,52^ including the submandibular gland.^53^ Furthermore, estrogen is an important factor in maintaining salivary flow.^54,55^ In this review, it was not possible to certify the effects of sex on salivary flow, due to the low number of studies presenting these values and the very low certainty of the evidence provided.

Despite the inclusion of a broad search strategy in several databases, including the gray literature, publication bias was evident. This method of analysis may have limitations when the studies present important methodological deficiencies.^19^ Studies with higher methodological quality and that control of confounding factors, such as age ranges and sex are necessary. Considering the implications of saliva as lubrication, protection, and maintenance of the balance of the oral microbiota for the health of patients with Down syndrome,^56,57^ preventive actions that reinforce oral hygiene, in view of the increased risk of periodontal diseases,^5,58^ are considered timely strategies in this population. Relative to dental caries, the results suggested that hyposalivation in patients with Down syndrome does not necessarily imply a higher number of dental caries cases.^30^

## Conclusion

With regard to the very low evidence, children and teenagers with Down syndrome have a lower salivary flow rate with an unstimulated saliva collection method compared with non-syndrome controls. Saliva collection methods can interfere with salivary flow analysis. The salivary flow rate can vary according to the age groups evaluated, generating high heterogeneity. In contrast, sex does not appear to interfere with salivary flow rate behavior.

## Data Availability

The authors declare that all data generated or analyzed during this study are included in this published article.
